# Switching from Multiplex to Multimodal Colorimetric Lateral Flow Immunosensor

**DOI:** 10.3390/s20226609

**Published:** 2020-11-18

**Authors:** Simone Cavalera, Fabio Di Nardo, Luca Forte, Francesca Marinoni, Matteo Chiarello, Claudio Baggiani, Laura Anfossi

**Affiliations:** 1 Department of Chemistry, Università degli Studi di Torino, 10124 Turin, Italy; simone.cavalera@unito.it (S.C.); fabio.dinardo@unito.it (F.D.N.); matteo.chiarello@unito.it (M.C.); claudio.baggiani@unito.it (C.B.); 2 PRIMA Lab SA, 6828 Balerna, Switzerland; lf@primahometest.com (L.F.); fma@primahometest.com (F.M.)

**Keywords:** point-of-care testing, gold nanoparticles, gold nanostars, HIV, serotyping

## Abstract

Multiplex lateral flow immunoassay (LFIA) is largely used for point-of-care testing to detect different pathogens or biomarkers in a single device. The increasing demand for multitargeting diagnostics requires multi-informative single tests. In this study, we demonstrated three strategies to upgrade standard multiplex LFIA to multimodal capacity. As a proof-of-concept, we applied the strategies to the differential diagnosis of Human Immunodeficiency Virus (HIV) infection, a widespread pathogen, for which conventional multiplex LFIA testing is well-established. In the new two-parameter LFIA (x^2^LFIA), we exploited color encoding, in which the binding of multiple targets occurs in one reactive band and the color of the probe reveals which one is present in the sample. By combining the sequential alignment of several reactive zones along the membrane of the LFIA strip and gold nanoparticles and gold nanostars for the differential visualization, in this demonstration, the x^2^LFIA can furnish information on HIV serotype and stage of infection in a single device. Three immunosensors were designed. The use of bioreagents as the capturing ligand anchored onto the membrane or as the detection ligand labelled with gold nanomaterials affected the performance of the x^2^LFIA. Higher detectability was achieved by the format involving the HIV-specific antigens as capturing agent and labelled secondary bioligands (anti-human immunoglobulins M and protein G) as the probes.

## 1. Introduction

The use of point-of-care test (POCT) has been extensively employed in developing countries, where laboratory settings are often unavailable [1]. The lateral flow immunoassay technique (LFIA) has been extensively employed for setting up POCT, as it meets all requirements established by WHO for this kind of tests [2]. Its application spans from healthcare to farming hazard assessment, by screening disease-relevant biomarkers, bacteria, viruses, toxins, contaminants and so forth [3,4]. In the last decade, the increasing demand for simultaneous detection of multiple biomarkers in a single assay, has resulted in the development of unnumbered multiplexed LFIAs [5,6,7,8]. In a typical multi-target LFIA, the number of information items corresponds to the number of test lines drawn on the strip. One of the main applications of LFIA is their use for diagnosing infectious diseases the multiplexing approach can be used for different purposes: serotyping [9], infection stage differentiation [10,11], discrimination between similar infections [12]. However, reacting bands cannot be increased endless and thus the number of information that can be obtain in a single test is limited to few [13,14]. Multiplexing approaches exploiting probes with tuneable signals have been reported, based on fluorescence and chemiluminescence encoding [15,16,17]. Nevertheless, these approaches need instrumentation that limits on-field applications. Dual-color probes, such as gold nanoparticles (GNP) and gold nanostars (GNS) [18] or silver nanoparticles (SNP) [19] functionalized with antibodies were used in previous studies to develop multiplexing LFIA in which the differentiation of the probe enabled distinguishing the target [18,20,21].

Here, we propose a two-parameter multiplexing LFIA strategy (x^2^LFIA), which combines multiple lines and the color-encoded approach to expand the number of information achievable within a single strip test. The work aims at demonstrating the feasibility and the potentiality of the x^2^LFIA approach to get a tetravalent information in a two-line and two-color assay.

**Figure 1:**
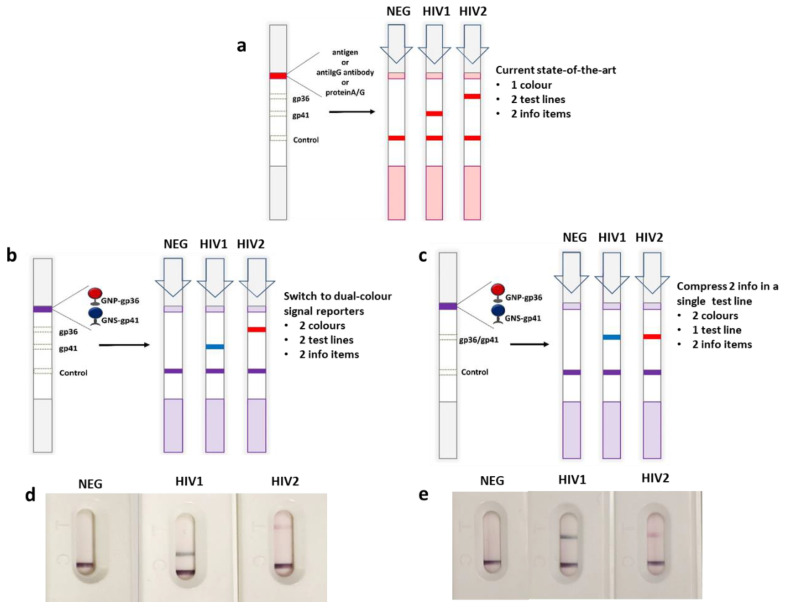
Conceptual framework of the new generation multiplexing LFIA with the addition of a dual-color probe: state-of-the-art in multiplexing (**a**) schematic of the possible use of red and blue colored gold nanomaterials to discriminate HIV1 and HIV2 (**b**) and to merge the two information items in a single test line (**c**). Results from a known negative, a HIV1 positive, and a HIV2 positive serum samples, respectively are also shown to confirm the feasibility of the xLFIA (**d**) and x^2^LFIA (**e**).

As a proof-of-concept of the multimodal approach, we used a set of immunoreagents for the diagnosis of the Human Immunodeficiency Virus (HIV) infection. HIV is one of the main fields of application of point-of-care testing since it still has a severe impact on society [22] and especially in low-resource settings. POCT diagnostics involving conventional and multiplex LFIA for HIV are well-established [23]. HIV testing rely typically on the detection of the serological response to the infection. In particular, antibody-screening LFIAs discriminate between HIV1 and HIV2 serotypes by exploiting the specificity of the recognition between the type-dependent viral proteins and the human anti-HIV antibodies [24]. Recombinant envelope glycoproteins gp41 and gp36 are generally used as the antigens to specifically recognize HIV1 and HIV2 antibodies, respectively [25,26]. The spatial resolution is the most common strategy to multi-targeting the single assay [27,28,29,30,31,32,33]. For instance, in a typical HIV serotyping test, the discrimination is made by coating the specific antigens in two spatially confined bands (test lines) (Figure 1a) and anti-HIV antibodies are indiscriminately revealed by labelled secondary antibodies as the signal reporters. The anti-HIV antibodies form immunocomplexes with the labelled secondary antibodies and are captured by the antigens at the test lines. This results in the accumulation of the signal reporters with the formation of colored lines. On the other hand, the double antigen approach has been reported also. In this case, the probe is represented by the labelled antigen and the sandwich-type complex comprises the anti-HIV antibody bridging two antigens, one anchored to the support (capture) and the second one labelled (detection). In microplate-based ELISA this approach was shown to increase specificity and sensitivity and some LFIA formats adopted this strategy [34,35].

The serological response to infection involves the production of different classes of immunoglobulins, which follows a typical temporal pattern, in which immunoglobulins M (IgM) are produced first, followed by immunoglobulins G (IgG) [36,37]. Then, the IgM/IgG ratio is exploited for identifying the stage of the infection [38,39]. HIV testing embedding both serotyping capability and infection stage discrimination are not available currently. We combined the two discrimination levels in a single test as the illustration of the potential of the multimodal approach.

Variously combining specific bioreagents as capturing agents coated onto the nitrocellulose and signal reporters conjugated to GNP and GNS two xLFIA and three x^2^LFIA immunosensors were designed and their performance investigated.

For the studying x^2^LFIA immunosensors and comparing their performance, we used gp41 and gp36 to bind specifically the two HIV serotypes and protein G and anti-human immunoglobulins M (anti-hIgM) secondary antibody to bind IgG and IgM, respectively. The three immunosensors differed each other by the role and position that immunoreagents played in the assay. Immunosensor 1 used secondary bioligands (e.g., protein G and anti-hIgM) as the capturing agents and the GNP/GNS -labelled HIV antigens as the probes. Immunosensor 2 was the exact reverse. The two HIV antigens were coated on the test lines and acted as the capturing agents while secondary bioligands were labelled with two distinct signal reporters. Immunosensor 3 relied on the double antigen strategy, where HIV antigens were used both for capture and for signal reporting, both linked to the red GNP. The blue-labelled anti-hIgM was added to add the information on infection stage. Generally, the double antigen approaches are reported as more sensitive than other formats, precisely because of its selectivity [13]. The three immunosensors were tested using a panel of control sera for investigating the ability to combine the spatial and color resolution to provide multiple response and for elucidating the effect of the role played by the immunoreagents on the immunosensors performance.

The importance of multiplexing is growing day by day, parallel to the comprehension of the relevance of intersecting information on several biomarkers at one time [40,41,42,43]. This study discloses the possibility to expand the multiplexing capability of the LFIA platform for multi-target screening tests without requiring expensive instrumentation for miniaturized spots reading.

## 2. Materials and Methods

### 2.1. Chemicals and Materials

Gold (III) chloride trihydrate (ACS reagent), hydroquinone, streptococcal protein G, sodium caseinate, anti-human IgM (µ chain specific) antibody produced in goat, and bovine serum albumin (BSA)were purchased from Sigma–Aldrich (St. Louis, MO, USA). Tween20 and other chemicals were of analytical grade and were obtained from VWR International (Milan, Italy). HIV-antigens gp36 and gp41 were purchased from Arista Biologicals Inc. (Allentown, PA, USA). Casein-biotin for conjugation to GNPs was obtained from In3diagnostics (Torino, Italy). Nitrocellulose membranes with cellulose adsorbent pad (CNPC-SS12-L3-P25) and sample pads (FR-1) were purchased from MDI membrane technologies (Ambala, India), while conjugate pads (GF) were obtained from Merck Millipore (Billerica, MA, USA).

### 2.2. Synthesis of Gold Nanoparticles and Gold Nanostars

Spherical gold nanoparticles (GNP) were synthesized by the usual tetrachloroauric acid reduction with sodium citrate [44]. GNP to be used as probes were obtained by adding 1.0 mL of sodium citrate (1%) to 100 mL boiling aqueous tetrachloroauric acid (0.01%, *w*/*v*) under vigorous stirring. GNS were synthesized through a seeding growth approach using a stepwise reduction of Au(III) to Au(I) by citrate and Au(I) to Au(0) by hydroquinone. The protocol followed one previously reported [20] and involved the synthesis of GNP seeds with a localized plasmon resonance (LSPR) band centered at 517 nm. These were obtained as described above, except for the volumes used, which were 0.6 mL of 1% *w*/*v* sodium citrate added to 30 mL of 0.01% tetrachloroauric acid.

For GNS preparation, 1.9 × 10^−8^ mol of tetrachloroauric acid was mixed with 9.3 × 10^−13^ mol of GNP seeds and 7.5 × 10^−9^ mol of sodium citrate. The mixture was stirred for 2 min at room temperature. Then, 3.0 × 10^−5^ mol of hydroquinone was rapidly under vigorous stirring. The solution was kept under stirring at room temperature for further 20 min.

### 2.3. Labelling Immunoreagents with GNPs and GNSs

**Figure 2:**
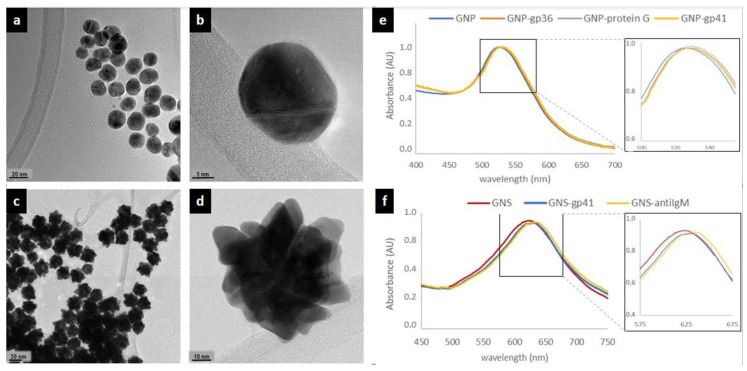
Spectroscopic characterization of gold nanomaterials: images obtained by high-resolution transmission electron microscopy of the GNP (**a**,**b**) and GNS (**c**,**d**), and visible spectra of bare GNP (**e**) and GNS (**f**) and of the conjugates to bioreagents. Insets show a magnification of the LSPR band region. Blue shifts witness increased dimensions due to the absorption of both the antigen and the saturation protein.

The red-colored GNP-gp36 conjugate was prepared by adsorbing the gp36 antigen onto the GNP surface. In detail, 10 mL of GNP (optical density ca. 1) was basified with carbonate buffer (50 mM pH 9.6) to pH 8 and added dropwise with 100 μg of gp36 antigen under gentle stirring at room temperature for 40 min. Next, 1 mL of casein (5% in borate buffer) was added and reacted for 10 min to saturate the free GNP surface. GNP-gp36 was recovered by centrifugation (8000× *g* 10 min) and washed with borate buffer supplemented with 0.5% casein. Finally, the GNP-gp36 was re-suspended in the CAS-storage buffer (borate buffer with 0.5% casein, 0.25% Tween 20, 2% sucrose and 0.02% sodium azide) and stored at 4 °C until use. The red GNP-gp41 and GNP-biotin conjugates were obtained by the same procedure. The blue-colored GNS-gp41 and GNS-anti IgM conjugates were prepared likewise, except that GNS with optical density ca 0.6 were used and reacted with 10 μg of protein. For the GNP-protein G conjugate, 10 mL of GNP was brought to pH 6 with carbonate buffer and added with 20 μg of protein G under gentle stirring at room temperature for 40 min. Next, 1 mL of BSA (10% in borate buffer) was added and reacted for 10 min to saturate the free GNP surface. GNP-protein G was recovered by centrifugation (8000× *g*, 10 min) and washed with borate buffer supplemented with 1% BSA Finally, the GNP-protein G was re-suspended in BSA-storage buffer (borate buffer with 1% BSA, 0.25% Tween 20, 2% sucrose and 0.02% sodium azide). Successful conjugation of proteins to gold nanomaterials was confirmed by UV-vis spectroscopy (Figure 2e,f).

### 2.4. LFIA Strip Preparation

The configurations of the preliminary LFIA immunosensors and of the three formats of x^2^LFIA are shown in Figure 1 and Figure 2. All strips were prepared by dispensing the immunoreagents on nitrocellulose membranes (CNPC-SS12-L3-P25) employing an XYZ3050 platform (Biodot, Irvine, CA, USA). Immunoreagents were diluted in phosphate buffer (20 mM, pH 7.4) to a concentration of 0.5 mg/mL and were dispensed at a flow rate of 1 μL/cm, keeping 4 mm between the lines. The concentration of 0.5 mg/mL was chosen as the best compromise between signal intensity and non-specific interaction with the GNP and GNS conjugates. After coating, membranes were dried at 37 °C for 60 min under vacuum. The conjugate pads were previously saturated with borate buffer supplemented with 0.25% Tween 20, 2% sucrose and 0.02% sodium azide, dipped into the proper probe solution at optimal optical density (2–3) and dried for 3 h at room temperature, protecting from light and dust. Strips were composed by layering the NC membrane with the sample and the conjugate pads and cutting (4 mm width) by CM4000 guillotine (Biodot, Irvine, CA, USA). Strips were finally included into plastic cassettes (Kinbio, Shangai, China) to obtain stand-alone LFIA devices.

### 2.5. Serum Samples

Control sera used in the study are listed in Table 1. Negative, HIV1 and HIV2 positive samples (#10, #8 and #5 respectively) and HIV1 seroconverting positive samples were from Zeptometrix International FDA approved HIV- panels. Samples X (9081-03), Y (9089-06) and Z (9019-03) were taken after 27, 26 and 38 days from infection, respectively.

### 2.6. The LFIA Test Procedure

Serum samples of the HIV panels were diluted 1/10 in the dilution buffer (phosphate 20 mM buffer, pH 7.4 with 1% BSA, 1% Tween20); 80 μL of the mixture was applied to LFD device and the results were visually inspected after 10 min.

The immunosensors were designed as qualitative ones, and the results were interpreted visually by comparing the color of the test lines with the one of the control lines to judge on the red/blue balance. However, to confirm visual judgements, we estimated red and blue color contribution to mixed lines by a RGB analysis, as detailed in Di Nardo et al. [18]. Briefly, the blue and red color channels were plotted as histograms and a threshold approach was applied to count the number of pixels for each channel. The R and B colors measured at the test line were normalized by the corresponding one measured at the control line.

## 3. Results and Discussion

### 3.1. Design of Multimodal LFIA Immunosensors

Preliminary, we investigated the applicability of the signal resolution obtained through labelling the specific recognition elements with dual color gold nanomaterials (e.g., gold nanospheres, GNP and gold nanostars, GNS). Gold nanomaterials were prepared as previously reported [18,20] and were characterized through transmission electron micrography and UV-vis absorption. GNP were almost spherical, mono-dispersed and with a mean diameter of ca. 30 nm (Figure 2a,b) The UV-vis spectrum showed a localized surface plasmon resonance (LSPR) band centered at 525 nm (Figure 2e), which corresponded to the perception of a ruby red color. GNS exhibited a blue color that was confirmed by the position of the LSPR band centered at ca. 620 nm (Figure 2f). Transmission electron microscopy images showed nanomaterials characterized by larger diameters (ca. 70 nm) and a star-like structure (Figure 2c,d).

We employed them in a conventional two-line serotyping strip based on the double antigen approach and labelled the HIV1 specific antigen (gp41) with GNS (blue) and the HIV2 specific antigen (gp36) with GNP (red), respectively (Figure 2e,f) to verify the functionality of the in-house prepared LFIA strips and of probes. The model LFIA was a rapid test for HIV1/2 antibodies detection developed by Primalab srl and under evaluation of the performance according to EC directive 2009/886 [45]. In the traditional format, the antigens were both labelled by GNP and deposed to form two test lines (Figure 1a). Selectivity and affinity of the labelled antigens towards HIV1/2 antibodies and their specificity towards other serum components (e.g., other proteins) were then assumed based on the performance of the traditional test.

To this aim, a protocol to prepare stable gold nanomaterials-antigen probes was established. In particular, the use of high amounts of casein (5%) in the saturation step stabilized GNP and GNS antigen conjugates and protected from non-specific interactions (see Supplementary Materials for further details). However, the use of casein to protect gold nanoprobes from aggregation, led to the partial inhibition of the specific interactions, as well. Finding a compromise in the use of casein was needed to equilibrate the S/N ratio.

The amount of capture bioreagents and probes was adjusted to reach clearly perceivable coloring at test lines (details on LFIA immunosensor setting up are reported in the Supplementary Materials). The blue GNS-gp41 and the red GNP-gp36 probes were mixed and included in the conventional xLFIA for HIV serotyping. An additional (red) GNP-biotin probe was added to form the signal at the control line, which comprised avidin. In fact, the setup of the double antigen approach includes an independent system to create the control line, not influenced by the specific probes, which can be regarded as a limitation of the strategy.

There was no mutual interference between the different signal reporters; no false positive signals were observed by applying the control negative sample, while HIV1 and HIV2 positive samples were correctly assigned on the basis of the position of the line and on the color of the probe (Figure 1b).

The mix of probes was then applied to a device with a single test line formed by a mix of the two antigens to compress the two information items in a single line (Figure 1c) and to achieve the signal resolution that we also have called “color-encoding” strategy [18]. If the stability and the absence of mutual interference was maintained no color change on respect to the two-line system was expected, since HIV-antibodies to gp36 and gp41 are known to do not cross-react with each other [46]. The absence of any false positive results due to non-specific binding and the correct assignment of the serotype based on the “color code” was verified in these conditions. The color code was defined as follows: a red coloring of the test line was interpreted as positivity associated to HIV2 serotype while the blue color indicated positivity again but due to HIV1 serotype, finally no coloring of the test line meant absence of any serological response and thus negativity (Figure 1c).

Based on the results from these preliminary studies, three different x^2^LFIA immunosensors were designed to disclose the potential of the multimodal strategy to combined multiple information in a single device. In this illustration, the prototypes allowed four information items to be obtained from two lines and two probes for the discrimination of the serotype (HIV1/HIV2) and of the class of immunoglobulins IgG/IgM) present in the patient’ serum. Therefore, we introduced two additional bioligands specific to IgM (anti-human IgM) and IgG (protein G), respectively. Two out of the three immunosensors varied for the role played by immunoreagents (capture or detection) in the typical serological immunometric assay and the third was designed to apply with the double antigen approach.

**Figure 3:**
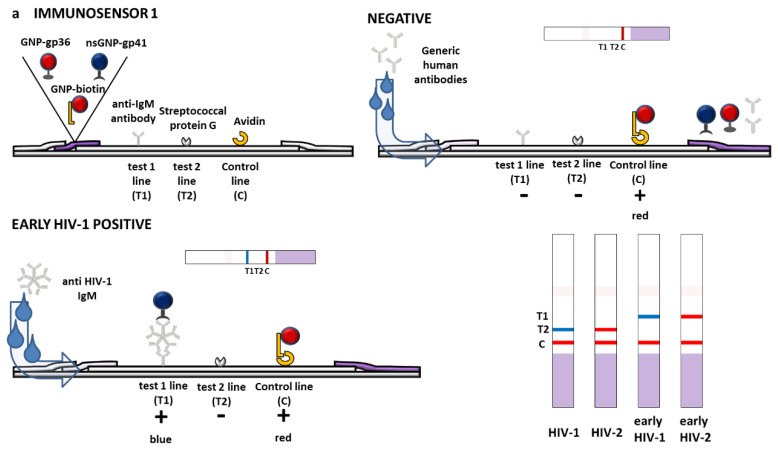

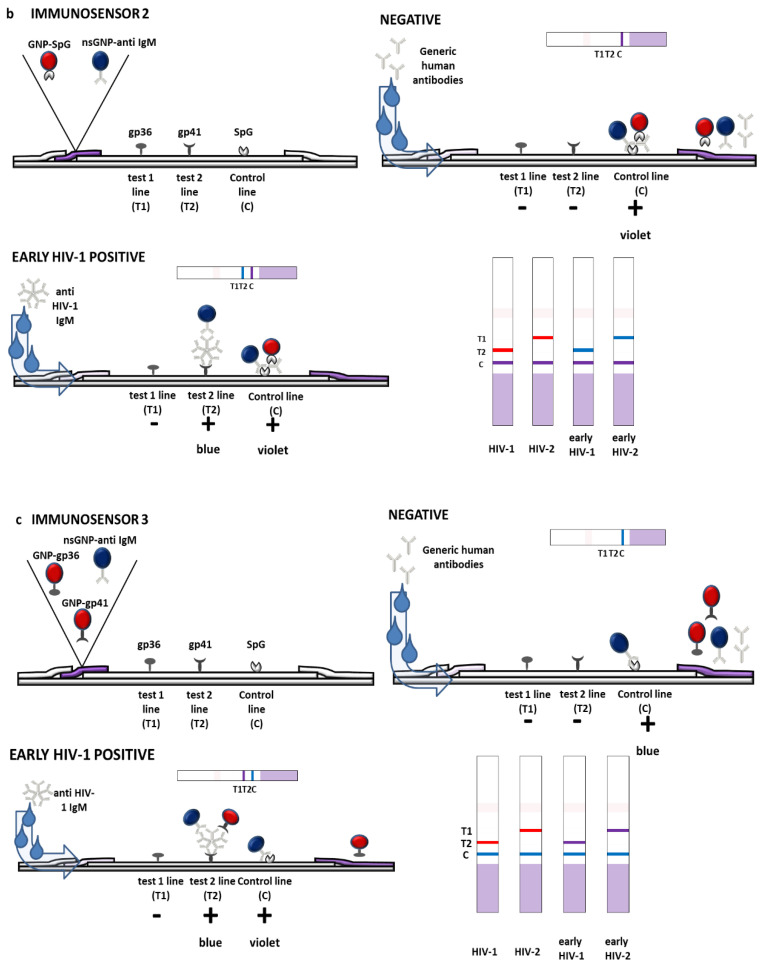
Scheme of the three immunosensors of x^2^LFIA unlocked by the combining spatial resolution with dual color gold nanomaterials. The binding and colored results expected for a negative and a HIV1 positive sample containing both IgG and IgM (early infected) is depicted. The three formats varied for the role played by immunoreagents as follows: (**a**) HIV-specific antigens were labelled and reacted with anti-HIV antibodies in the sample, which were then captured by anti-hIgM and protein G coated to form test lines (immunosensor 1); (**b**) HIV-specific antigens were coated and captured anti-HIV antibodies, which were revealed by labelled anti-hIgM and protein G (immunosensor 2); and (**c**) HIV-specific antigens were both coated and labelled and reacted with anti-HIV antibodies to form a double antigen sandwich. The addition of the blue-labelled anti-hIgM to the red-labelled antigen provided the additional information on the infection stage (immunosensor 3).

The format of immunosensor 1 included secondary ligands (anti-human IgM and protein G) separately coated on two test lines to capture anti-HIV antibodies. Therefore, the spatial resolution allowed for discriminating the immunoglobulin class. The differentiation of the serotype was realized on each line by employing the two signal reporters, namely GNS-gp41 to reveal HIV1 and GNP-gp36 to reveal HIV2, respectively. The GNP-biotin probe was added to form the control line (Figure 3a, Immunosensor 1). In the second immunosensor, the role of capturing and detection reagents was inverted. Hence, the first gp36-coated test line captured anti-HIV2 antibodies and the second gp41-coated test line captures anti-HIV1 ones. By using (blue) GNS-anti-IgM antibody and (red) GNP-protein G as the signal reporters, we expected a blue response for very early infection and a progressive red shift proportional to seroconversion rate on each test line. (Figure 3b, immunosensor 2). In this secondo format, the control line comprised protein G, which was able to capture both probes and then directly reflected their stability.

The set-up of coated antigens described for immunosensor 2 was maintained for designing immunosensor 3. Instead, the mix of signal reporters included the two antigens both labelled with GNP (red) and the anti-human IgM labelled with blue GNS.

The total antibody response was red colored such as in a conventional double-antigen assay based on the two-line set-up. However, the presence of specific IgM turned the color of the line to violet, due to accumulation of the blue GNS-anti-human IgM probes. (Figure 3c, Immunosensor 3).

### 3.2. Performance of the x*^2^*LFIA Immunosensors for HIV Serotyping and Discrimination of the Infection Stage

To investigate the multiplexing capability of the three multimodal approaches and to compare their performance, the x^2^LFIA formats were tested by control human sera (Table 1). Results were visually observed and captured by common smartphone cameras. The images were processed and compared as relative intensities of the red and blue components of the colorimetric signal. The comparative study on immunosensors 1 and 2, which differed for the role played by immunoreagents, and with immunosensor 3, which differed for the assay format, were investigated in terms of detectability and specificity in correlation to what is expected by the control samples.

Details on the immunosensors format and on interpretation of the results are reported in Table 2.

**Figure 4:**
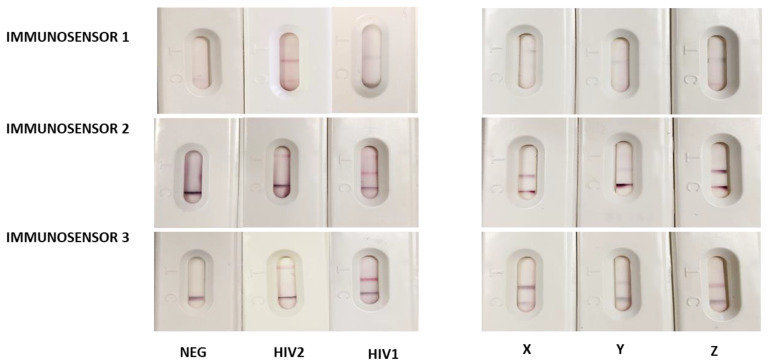
Multiple information obtained from the x^2^LFIA immunosensors on the negative (NEG), two fully seroconverted (HIV1 and HIV2) and the three early infected (X, Y and Z) serum samples from Zeptometrix panels.

The three x^2^LFIA formats were tested with control human sera and with samples from seroconversion panels, which were supposed to contain anti-HIV1 IgM. The outcomes considerably differed among the immunosensor formats, both in the color encoding response and in terms of detectability (Figure 4). All x^2^LFIA immunosensors correctly assigned the fully seroconverted positive samples and did not show non-specific binding with the negative sample. Immunosensor 1 provided a single test line at the T1 (IgM) or at the T2 (IgG) position, blue colored for HIV1 positive and red colored for HIV2 positive sera, respectively. The seroconverting samples resulted in two blue-colored test lines, where the variable IgM/IgG ratio reflected in the relative intensities of the two lines (Figure 4, immunosensor 1 and 5a). Samples X and Y showed coloring at both lines, with a slight disproportion in favour of T1 (IgM) for sample X. On the contrary, sample Z showed an evident preponderance of IgG.

**Figure 5:**
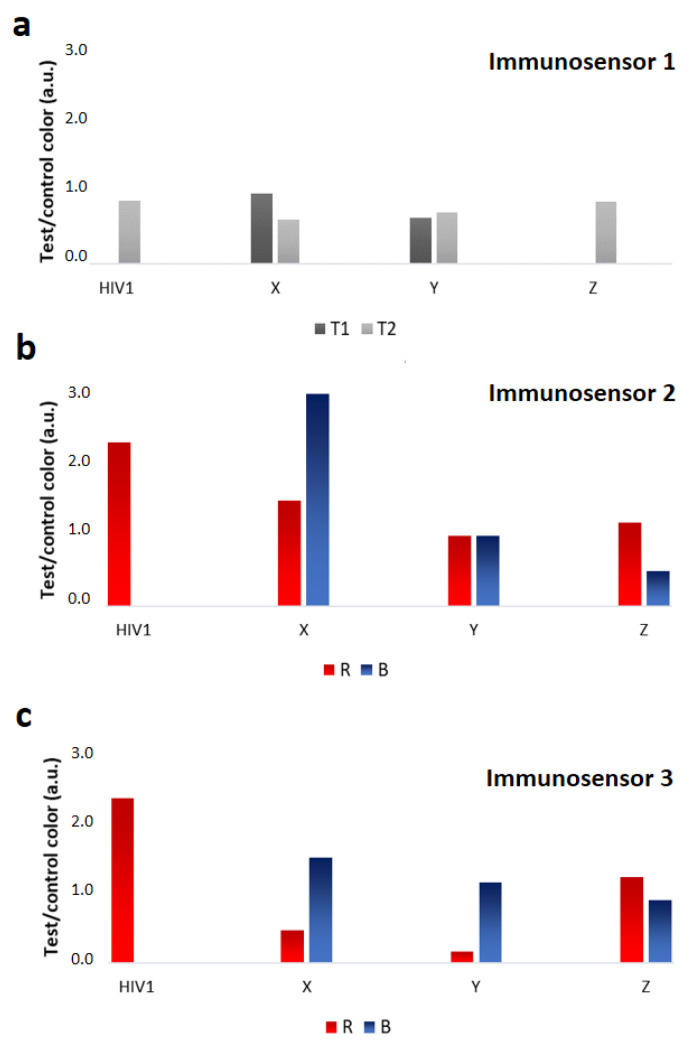
Color intensities for test and control lines were measured as the total number of pixels for Format 1 and reported as a function of line positioning (**a**). Red (R) and blue (B) components were extracted and measured for Formats 2 and 3 as reported in Di Nardo et al. [18] (**b**,**c**). Color at test lines was normalized for the corresponding one measured at the control line. Analysis were repeated in triplicate and mean RSD% were calculated between 4 and 16%.

In general, largely lower intensities were obtained from immunosensor 1 on respect to other two x^2^LFIA formats. Immunosensor 2 provided a red colored test line at T2 position for HIV1 positive, and at T1 position for HIV2 positive sera, respectively. The three seroconverting samples resulted in a single violet test line at the T2 (HIV1) position, which intensity varied (Figure 4, immunosensor 2). Serum X provided a signal at the test with a larger contribute of the blue probe compared to the control line indicating the prevalence of blue-labelled anti-hIgM, while serum Z showed a red-purple color at the test line, witnessing the prevalence of the red GNP-protein G reporter (Figure 5b). Compared to immunosensor 1, using anti-hIgM antibody and protein G for signal reporting and the antigens as capturing agents instead of the opposite increased the detectability (Figure 4). We argued that the difference on the fact that probes disposed of longer time to bind to their targets (because they are mixed with the sample and reacts during flowing), while capturing reagents should be particularly efficient as the time of contact with the target was limited. Alternatively, we guessed that the different flow rate of the HIV-specific antibodies compared to non-specific immunoglobulins present in the serum samples determined the observed behavior. In format 1, anti-HIV antibodies bound to the GNP-labelled antigen and were slower than unbound non-specific antibodies. Probably, the faster unbound immunoglobulins reached the lines of capturing reagents first so inhibiting the following binding of the specific ones linked to GNPs. In immunosensor 2 all immunoglobulins bound to GNP probes and moved with the same velocity, while immunosensor 3 eliminated the competing binding of non-specific immunoglobulins, as it included only HIV-specific bioreagents. Immunosensor 1 and 2 furnished spatially separated outcomes, as a “two-line x two-color” response. Immunosensor 3 showed a single red colored test line, at the T2 position for HIV1 positive and at the T1 position for HIV2 positive sera, respectively. In the absence of anti-HIV IgM, the results from the immunosensor 3 overlapped the ones from immunosensor 2. Serum belonging to individuals with late infections (HIV1 and HIV2) provided a response indicating the sole presence of immunoglobulins G (Figure 4, immunosensor 3). Compared to immunosensor 2, the signal intensity was almost unaffected by the change of the probes. The sensitivity was supposed to be boosted by the double antigen approach; however, we did not observe relevant improvements, except on serum Y. The presence of the blue GNS-anti-hIgM antibody provoked a violet-shift in the presence of anti-HIV IgM, because of the additive effect of red and blue probes. The three seroconverting samples resulted in a single intense violet test line at the T2 (HIV1) position. Patient Z resulted in an almost red line, suggesting the prevalence of IgG, while sample X and Y provided a blue-shifted line, which indicates the presence of IgM, besides IgG (Figure 5c).

The classification of the three seroconverting sera (X, Y, and Z) was coherent within the immunosensor formats. Anti-HIV1 IgM significantly contributed to the serological response for patients X and Y indicating very early infection, while patient Z was assigned as having predominantly IgG, though some IgM were also revealed.

The outputs of x^2^LFIA were in agreement with the time of blood collection from infection, as well. Serum Z (9019-03) was a sample taken 38 days from infection, while the other two were from earlier sampling (27, 26 days). The color evaluation, though susceptible of subjective interpretation was facilitated by the comparison to the control line, which acted as a sort of internal reference, besides confirming the validity of the assay, as usual. Immunosensor 3 appeared to be more sensitive to IgM variation in a narrow interval compared to others. Noticeably, immunosensor 3 added the discrimination ability by simply including an additional probe with differentiable signal to the standard double antigen set-up.

Multiplexing LFIA based on differentiable color probes are conventionally based on using latex microparticles embedding dyes with different adsorption peaks [47,48] and noble metal nanoparticles showing localized plasmon resonance bands at variable wavelengths according to their size and shapes [17,20,21].However, these approaches involves aligning several lines along the strip to differentiate the target to be detected and the color of the probe is simply exploited to help simplifying the visual reading of the result. Here, we designed a two-parameter strategy, in which the color and space resolution were combined to expand the number of analytes simultaneously detected by a single device. As a proof-of-concept, dual color probes (i.e., red and blue gold nanomaterials) were combined with a two-line arrangement of capturing ligands to reach a 2 × 2 analytical platform (x^2^LFIA). Theoretically, the strategy could be implemented by including more than two different probes and by aligning more than two test line. In this regards, the use of latex microspheres embedding dyes will increase further the number of information that could be furnished by a single device.

## 4. Conclusions

The use of LFIA devices for HIV diagnosis is well-established and largely diffuse, as such we use it as the model as a proof-of-concept to verify the feasibility of the multimodal approach combining the spatial resolution with color encoding to expand the multiplexing capability of the LFIA platform. Taking advantage of the unique spectroscopic properties of gold nanomaterials and the simplicity of their conjugation with proteins (antigens and antibodies) by passive adsorption, three x^2^LFIA immunosensors were designed. Immunosensors were explored in this work, to investigate the ability of differentiating serum samples belonging to individuals with different serological conditions. We investigated, also, the impact of changing the role of immunoreagents, in the x^2^LFIA set-up, showing that it strongly affected the detectability of the assay. Moreover, we designed a strategy enabling the discrimination of the antibody class that can be embedded in the conventional double antigen strategy.

In conclusion, we introduced a general route to enlarge the number of information achievable within one LFIA strip as the product of the number of probes for the number of lines, conserving the one-step and equipment-free operability.

## Figures and Tables

**Table 1:** The human serum samples from panels used in the study: one negative (#10), two fully seroconverted samples (#8 and #5) and 3 early-infected HIV1 positive samples.

ID (#)	Serotype	Type Elapsed from Infection (Days)	Seroconversion
NEG (#10)	Negative	-	-
HIV1 (#8)	HIV1	>90	complete
HIV2 (#5)	HIV2	>90	complete
X (9081-03)	HIV1	27	In progress
Y (9089-06)	HIV1	26	In progress
Z (9019-03)	HIV1	38	In progress

**Table 2:** Schematic of the xLFIA and x^2^LFIA formats used in this study.

		Reporter				Capture		
Format		Adsorbed on GNP		Adsorbed on GNS	Optical Density (Ratio) ^b^	Test Line 1 (T1)	Test Line 2 (T2)	Control Line (C)
xLFIA	A	gp36	biotin ^a^	gp41	2.5 (1 + 0.5 + 1)	gp36	gp41	avidin
	B	gp36	biotin ^a^	gp41	2.5 (1 + 0.5 + 1)	gp36/gp41	-	avidin
x^2^LFIA	1	gp36	biotin ^a^	gp41	2.5 (1 + 0.5 + 1)	anti-IgM	protein G	avidin
	2	protein G		anti-IgM	3 (1.5 + 1.5)	gp36	gp41	protein G
	3	gp36	gp41	anti-IgM	3 (1 + 1 + 1)	gp36	gp41	protein G

^a^ to form the control line, ^b^ the probes were mixed to reach the optical density in variable ratio.

## Supplementary Materials

The following are available online at https://www.mdpi.com/1424-8220/20/22/6609/s1, Electronic Supplementary Information (ESI) available: Further details about: (i) Study on the stabilization of GNS-gp41 conjugate, (ii) Development of the x^2^LFIA immunosensors, (iii) Preliminary interface study for GNP and GNS conjugates. Includes Table S1: Experimental conditions tested for the adsorption of the recombinant antigen onto GNSs.
