# Supplementary material for: Switching from Multiplex to Multimodal Colorimetric Lateral Flow Immunosensor
Source: Sensors (Basel). 2020 Nov 18;20(22):6609. doi: 10.3390/s20226609 (PMC7698945; doi:10.3390/s20226609)
Supplement: Supplementary file 1 [file sensors-20-06609-s001.pdf]

Switching from Multiplex to Multimodal Colourimetric Lateral Flow Immunosensor

Simone Cavalera[a], Fabio Di Nardo[a], Luca Forte[b], Francesca Marinoni[b], Matteo Chiarello[a], Claudio Baggiani[a] and Laura Anfossi*[a]

[a] *Department of Chemistry, University of Turin, Turin (TO), Italy*
[b] *PRIMA Lab SA, Balerna, Switzerland*

Supplementary Information

Preliminary interface study for GNP and GNS conjugates……………………………………………………………………………1
*Preparation and stabilization of GNS-gp41 conjugate*1
Table S1. Experimental conditions tested for the adsorption of the recombinant antigen onto GNSs.. ... 2~~3~~

Preliminary study for GNP and GNS conjugates preparation

Generally, previously described flocculation test is a common preliminary phase to estimate the minimum stabilizing concentration of protein to functionalise the GNP and GNS. However, antigens are not always suitable for such studies, being less robust than antibodies and less able to protect the GNP from aggregation. In fact, no info was made available from the flocculation tests about the adsorption of the gp36 antigen onto the red GNP and of the gp41 onto the GNS. For biotin and gp36 adsorption on the GNP and for the anti-M antibody adsorption on the GNS 4 to 10 µg of protein per ml*OD of GNP resulted widely sufficient to passivate the gold surface. Protein G showed protection from aggregation at as low amount as 2 µg/ml*OD. For this work we decided to normalize to 10 µg/ml*OD all the conjugates (with exception for multivalent protein G, 2 µg/ml*OD).

Preparation and stabilization of GNS-gp41 conjugate

Studies aimed at reaching stable and functional GNS-gp41 conjugate were conducted by varying the additive used to saturate the free GNP surface and the pre-saturation of the conjugate pad onto which the conjugate was dispensed (Table S1). Optimal conditions were established on a pass-no pass approach based upon i) avoiding GNP agglomeration during and after the conjugation process, ii) providing no signal for negative samples, and iii) showing intense colouring with positive samples. For the experiments, 3 serum samples #5 (HIV2+), #8 (HIV1+) and #10 (negative) from Zeptometrix panel were tested. The evaluation was made by naked eye observation between 5 and 10 minutes from sample application. Symbols represent: (-) absent, (-/+) weak, (+) moderate, and (++) strong red-colour developed at the test lines. The performance of the GNS-gp41 was considered acceptable if the agglomeration tendency was

negligible (pass), the signal for negative sample was absent/weak (pass) and the signal of positive samples from moderate to strong (pass). GNS-gp41 adsorption was carried at room temperature under gentle stirring. Stable GNS-gp41 conjugates were obtained by using 10 µg/ml*OD of gp41 to GNP at pH 8 with casein in the overcoating, washing and storage buffers.

Typically, the colour of the solution turned dark brown during the conjugation step, due to the hydroquinone shift in basic medium. After washing, hydroquinone was eliminated, and the colour returned blue and remained stable hereafter. The GNS-*gp41* conjugate did not show any aggregation phenomena, which should be expected for unstable antigen gold-conjugates. Results on the preliminary LFIA are shown in figure S1.

Table S1. Experimental conditions tested for the adsorption of the recombinant antigen onto GNS. Selected conditions for the preparation of GNS-*gp41* conjugate are shown in bold. The saturation agents were dissolved in borate buffer. The same buffer was used in the saturation, washing and resuspension steps of the conjugation. Unsuitable conditions were no further investigated (nd means that previous check was not passed)

**Saturation**		**Agglomeration tendency**	**Pre-adsorbed agent**		**Signal for negative sample (NEG)**	**Signal for positive sample (HIV1+)**
**agent**	**concentration (% w/v final)**		**agent**	**concentration (% w/v final)**		
BSA	0.1	moderate	nd	nd	nd	nd
BSA	1	weak	nd	nd	nd	nd
**Casein**	**0.5**	**no**	**none**	**nd**	**-**	**++**
Casein	0.5	no	BSA	1	+/-	nd
Casein	0.5	no	casein	0.5	-	+
